# The Role of Relationship Biography in Advance Care Planning Among Older Adults

**DOI:** 10.1093/geroni/igaf122.148

**Published:** 2025-12-31

**Authors:** Zhe Zhang, Lucie Kalousova

**Affiliations:** Vanderbilt University, Nashville, Tennessee, United States; Vanderbilt University, Nashville, Tennessee, United States

## Abstract

As individuals approach end-of-life, advance care planning (ACP) becomes a critical aspect of personal and familial well-being. Prior research on ACP focuses on static marital status—such as whether an individual is currently married, divorced, or widowed—as predictors of ACP behaviors. However, this approach overlooks the cumulative and dynamic nature of relationship histories, leaving a critical gap in understanding how one’s full relationship trajectory shapes end-of-life decision-making. Guided by a gendered life course perspective, this study addresses this gap by examining how relationship trajectories shape three ACP dimensions: living wills, durable power of attorney for healthcare (DPAHC), and end-of-life discussions. Using data from the 2020 Health and Retirement Study (HRS), a nationally representative longitudinal survey of older Americans, we trace individuals’ relationship histories and assess their associations with ACP behaviors, with attention to gender differences. Multivariable logistic regression results show that, compared to those continuously married, older adults who are partnered, widowed, or unmarried after multiple disruptions are more likely to have a living will. Similarly, the divorced, widowed, and those unmarried after two or more disruptions have higher odds of naming a DPAHC. For end-of-life discussions, only one group significantly differs from the continuously married: individuals remarried after two or more disruptions have higher odds of having these conversations. We detect modest gender differences in these patterns. This study advances research on the social determinants of ACP and life course perspectives by demonstrating that relationship histories play a crucial role in shaping individuals’ preparedness for life’s final chapter.

